# Effect of Simulated Gastrointestinal Tract Conditions on Survivability of Probiotic Bacteria Present in Commercial Preparations

**DOI:** 10.3390/ijerph18031108

**Published:** 2021-01-27

**Authors:** Lidia Stasiak-Różańska, Anna Berthold-Pluta, Antoni Stanisław Pluta, Krzysztof Dasiewicz, Monika Garbowska

**Affiliations:** Department of Food Technology and Assessment, Institute of Food Sciences, Warsaw University of Life Sciences-SGGW, Nowoursynowska St. 166, 02-787 Warsaw, Poland; anna_berthold@sggw.edu.pl (A.B.-P.); antoni_pluta@sggw.edu.pl (A.S.P.); krzysztof_dasiewicz@sggw.edu.pl (K.D.); monika_garbowska@sggw.edu.pl (M.G.)

**Keywords:** probiotics, resistance, survivability, gastrointestinal passage, gut

## Abstract

Probiotics are recommended, among others, in the diet of children who are under antibiotic therapy, or that suffer from food allergies or travel diarrhea, etc. In the case of toddlers taking probiotic preparations, it is highly recommended to first remove the special capsule, which normally protects probiotic strains against hard conditions in the gastrointestinal tract. Otherwise, the toddler may choke. This removal can impair probiotic survival and reduce its efficacy in a toddler’s organism. The aim of this study was to evaluate the survivability of five strains of lactic acid bacteria from the commercial probiotics available on the Polish market under simulated conditions of the gastrointestinal tract. Five probiotics (each including one of these strains: *Bifidobacterium* BB-12, *Lactobacillus (Lb.) rhamnosus* GG, *Lb. casei*, *Lb. acidophilus*, *Lb. plantarum*) were protective capsule deprived, added in a food matrix (chicken–vegetable soup) and subjected under simulated conditions of the gastric and gastrointestinal passage. Strain survivability and possibility to growth were evaluated. Obtained results showed that, among all analyzed commercial probiotic strains, the *Lb. plantarum* was the most resistant to the applied conditions of the culture medium. They showed a noticeable growth under both in vitro gastric conditions at pH 4.0 and 5.0, as well as in vitro intestinal conditions at all tested concentrations of bile salts.

## 1. Introduction

The definition of “probiotic” provided by the International Scientific Association of Probiotics and Prebiotics states that probiotics are “live microorganisms that, when administered in adequate amounts, confer a health benefit on the host” [1]. The best known and the most thoroughly investigated probiotic strains are representatives of *Lactobacillus* (*Lb.*) and *Bifidobacterium* genera [2,3,4]. The role of probiotics is to, i.a., alleviate symptoms of lactose intolerance, ameliorate outcomes of food allergies, and reduce cholesterol concentration in blood [5,6,7]. The administration of probiotic preparations is recommended, e.g., during and after antibiotic therapy to aid the reconstruction of natural enteric microflora [8,9,10]. 

It is estimated that from 11 to 30% of children treated with antibiotics (mainly β-lactam ones and vancomycin) suffer from intestinal discomfort and diarrheas [11,12,13]. Diarrhea is especially dangerous for small children/toddlers as it may cause malfunction of the water–electrolyte balance of their bodies within a short period of time [14]. Sometimes, however, children suffer from post-antibiotic diarrhea despite their diet supplementation with probiotic strains [15]. This is, probably, caused by reduced survivability of individual probiotic strains under varying conditions of the alimentary tract. Oral administration of at least 10^7^ cells of a probiotic strain per milliliter or gram of food should ensure a positive effect to the host even when some of them do not survive the unfavorable conditions of the gastrointestinal passage [16]. Most of the commercial probiotics are registered as “dietary supplements” and therefore do not have to comply with quality requirements obligatory for drugs [17]. After oral administration, the probiotic strains are exposed to low pH of the stomach and bile salts in the enteral section of the alimentary tract of the host. Many of them often fail to survive conditions of the passage [18,19]. Metabolic and biochemical activity of probiotics during gastrointestinal passage can be sustained through earlier encapsulation of their cells [20,21]. However, probiotic preparation producers recommend removal of the protective capsule before giving it to toddlers and making a suspension of probiotic powder with water to avoid choking or strangulation during swallow. Such information can be found on preparation leaflets. Another means of protecting probiotics against adverse effects of the gastrointestinal conditions is their administration together with a prebiotic [22]. The best known and the most commonly used prebiotics include inulin and oligofructose [23].

The aim of this study was to examine the survivability and possibility to growth of strains obtained from commercial probiotic preparations (without protective capsule) under conditions simulating gastrointestinal tract.

## 2. Materials and Methods

### 2.1. Commercial Probiotic Preparations

The study was conducted with 5 commercial preparations, available on the Polish market, which contained lyophilized cells of one-strain of probiotic bacteria, namely:

Preparation 1—*Lactobacillus rhamnosus* GG ATCC 53103 (3 × 10^9^ CFU/one dose, 4.5 × 10^9^ CFU/g);

Preparation 2—*Bifidobacterium* BB-12 (4 × 10^9^ CFU/one dose, 1.7 × 10^10^ CFU/g);

Preparation 3—*Lactobacillus casei* (4 × 10^8^ CFU/one dose, 2.1 × 10^9^ CFU/g); 

Preparation 4—*Lactobacillus acidophilus* (2 × 10^9^ CFU/one dose, 3.1 × 10^10^ CFU/g); 

Preparation 5—*Lactobacillus plantarum* (4 × 10^8^ CFU/one dose, 1.8 × 10^9^ CFU/g). 

In the case of preparations 3, 4 and 5, producers did not provide any information about the number of strain or its origin. This information is a trade secret. Preparation 2 contained a prebiotic in the form of fructooligosaccharide (FOS), whereas preparations 3 and 4 contained inulin, and preparations 1 and 5 did not contain prebiotic. The preparations originated from various Polish producers and were registered as dietary supplements. Their production dates were similar. Preparation 1 was in the form of a lyophilizate in a paper sachet, whereas the other preparations were encapsulated in gelatin capsules. Preparation 1 was poured out of the sachet prior to testing. All gelatin capsules (which normally protect probiotics from outside the GIT environment) from preparations 2, 3, 4, and 5 were removed before using probiotic preparations in experiments; this is typically the intake procedure for probiotic preparations for toddlers (12–18 months) to avoid choking. 

### 2.2. Growth Media and Solutions

MRS broth (Sigma-Aldrich, St. Louis, MO, USA), agar 15 gL^−1^ (for seeding step), pH 6.2. The broth was sterilized at a temperature of 121 °C for 15 min.

Gastric electrolyte solution (GES) [24,25], composed of [gL^−1^]: NaCl 4.8, NaHCO_3_ 1.56, KCl 2.2, CaCl_2_ 0.22, pepsin 1. The solution was sterilized at a temperature of 121 °C for 20 min; after sterilization GES was supplemented with a filter sterile pepsin solution in water (P6887; Sigma-Aldrich, 0.22 μL, Sartorius Poland Sp. z o o.) to final concentration 1 gL^−1^.

Double-concentrated J broth (2 × JB) [24], was composed of [gL^−1^]: peptone 10, yeast extract 30, K_2_HPO_4_ 6, glucose 4. The broth was sterilized at a temperature of 121 °C for 20 min. Glucose solution was filtered (filter pore diameter—0.22 μm, Sartorius Poland Sp. z o o.) and added to 2 × JB after sterilization. Bile salts were subjected to mild sterilization (117 °C, 10 min) and added to sterilized 2 × JB. Concentration of bile salts (Sigma-Aldrich, B8631) was adjusted to 1%, 2%, and 3%.

Chicken–vegetable soup (CVS) composed of [gL^−1^]: chicken breast fillet 200, onion 35, carrot 100, celery root 30, and parsley root 65. The CVS was prepared in a Termomix Vorwerc cooker, at a temperature of 100 °C, for 60 min, with a mixing rate knob in position 1. The CVS was filtered (filter pore diameter—0.45 μm).

Spring water was recommended for small children, sterilized at temperature 121 °C for 20 min.

*Lactobacillus* and *Bifidobacterium* BB-12 were enumerated by the pour plate technique on MRS and MRS modified by adding 0.2% (*w*/*v*) lithium chloride and 0.3% (*w*/*v*) sodium propionate (MRS-LP), respectively [26].

### 2.3. Study Design and Culture Conditions

The experiment was divided into three stages: control cultures, stomach stage, and gastrointestinal stage.

To observe how tested bacteria react in optimal conditions (control), *Lactobacillus* was incubated in MRS broth, while *Bifidobacterium* on MRS-LP, deemed optimal for their growth, was adjusted to pH 6.2 either with HCl 5M or with NaOH 1M at a temperature of 37 °C, for 48 h. In order to limit the access of oxygen to the *Bifidobacterium*, cultures were carried out without shaking and, additionally, the access of air was cut off with a layer of water agar. Incubations in MRS adjusted to pH 2.0, 3.0, 4.0 or 5.0 in the same growth conditions were also performed to have a positive control of probiotics growth. In the tested preparations, the initial number of viable cells of the probiotic strain was initially determined by inoculating petri dishes with MRS medium (preparations 1, 3, 4 and 5, respectively) and with MRS-LP Agar medium (preparation 2). The obtained results were expressed as CFU/g of each preparation (2.1.)

**Figure 1:**
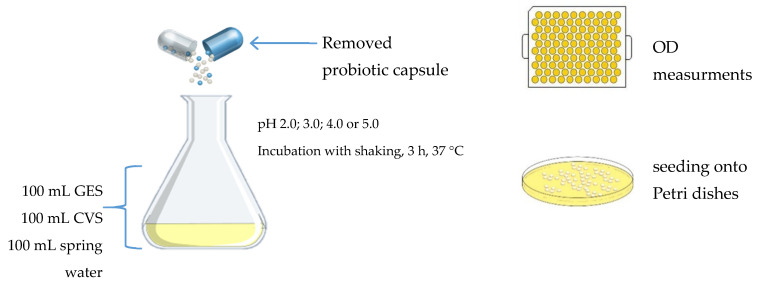
Scheme of the variants tested under gastric stage of experiment (GES—gastric electrolyte solution, CVS—chicken–vegetable soup).

To simulate conditions occurring in the stomach, 100 mL of GES was mixed with 100 mL of CVS and 100 mL of sterile spring water containing 1 dose of a given probiotic preparation (1, 2, 3, 4 or 5). Each of the five mixtures was cultured at final pH 2.0, 3.0, 4.0, and 5.0; temperature 37 °C, for 3 h (Figure 1). The acidity of the solutions was adjusted using the HCl 5M.

**Figure 2:**
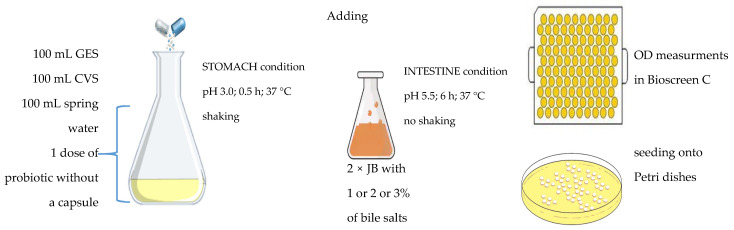
Scheme of the variants tested under gastrointestinal stage of experiment, (GES—gastric electrolyte solution, CVS—chicken–vegetable soup, 2 × JB—2 × concentrated J broth).

The gastrointestinal stage consisted of mixing 100 mL of GES with 100 mL of CVS and 100 mL of spring water suspension containing a given probiotic, at final pH 3.0. The mixture was shaken in a reciprocating shaker 50 rpm, at 37 °C for 30 min (stomach stage) [27]. Afterwards, the suspension was mixed with 2 × JB (1:1, *v*/*v*). Survivability of all preparations was examined in the presence of bile salts with concentrations of 1, 2 or 3 (%), at 37 °C for 6 h; the final pH of mixture medium was 5.5 (regulated using NaOH 1M) (Figure 2).

Optical density (OD) of individual cultures of probiotic preparations was measured every 30 min in a Bioscreen C MBR apparatus with the length of wave λ = 600 nm. Each culture variant was conducted in three independent replications. Simultaneously the count of bacteria on MRS Agar pH 6.3 (*Lactobacillus*) and MRS-LP Agar pH 6.3 (BB-12) plates was determined. Incubation was provided in anaerobic jars containing AnaeroGen^®^ (Argenta, Poland) [26]. The samples for spread on petri dishes was taken from time points: 0; 12; 24; 36 and 48 h for MRS conditions, 0; 1; 2; 3 h for gastric stage, 0; 2; 4; 6 h for gastrointestinal stage. In each variant of the experiment, three independent series of replicates were performed for each analyzed sample inoculated into petri dishes, differentiated by the type of preparation (1; 2; 3; 4; 5), variant type (MRS control, stomach, gastrointestinal (GI), pH (2.0; 3.0; 4.0; 5.0 or 6.2) or bile salt concentration (1%; 2%; 3%) and measuring point (h) 0; 12; 24; 36; 48 for MRS stage, 0; 1; 2; 3 for stomach, 0; 2; 4; 6 for GI). In the case of the optical density (OD) measurement in Bioscreen Apparatus, measurements were made every 30 min for each individual sample, and each sample variant was performed in three independent measurement series.

### 2.4. Calculation of Coefficient of Specific Growth Rate

The coefficient of the specific growth rate (μ) in time (t) was calculated from the formula: μ(t) = (ln OD_f_ − ln OD_i_) / (t_f_ − t_i_), where: OD_f_—final OD in the log phase, OD_i_—initial OD in the log phase, t_f_—time of log phase termination, t_i_—time of log phase onset [28]. 

### 2.5. Statistical Analysis

Results obtained were subjected to statistical analysis using StatGraphicPlus 4.1. software Statgraphics Centurion software (Version 17.1.12, Gambit Centrum Oprogramowania i Szkoleń Ltd., Kraków, Poland). Analysis of variance (ANOVA) was conducted. The Tukey test was applied to compare the significance of differences between mean values at a significance level of α = 0.05. Mean values from three replicates (*n* = 3) were also calculated, and the standard deviation was added to the mean as ± SD.

## 3. Results

### 3.1. Survivability and Growth of Bacteria Present in Commercial Probiotic Preparations in MRS Broth

Bacteria were cultured in the MRS broth deemed optimal for the growth of LAB and bifidobacteria [29,30,31] to observe how they react in optimal conditions. 

**Figure 3:**
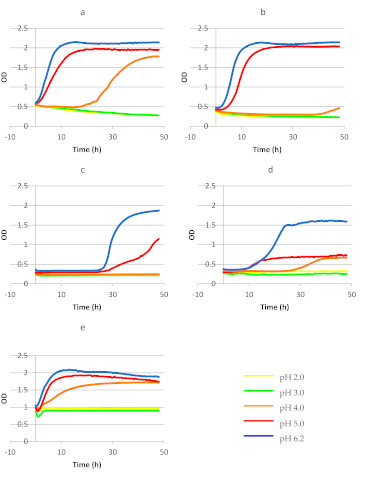
Changes in the optical density (OD_600_) during culture of commercial probiotics preparations: (**a**) *Lb. rhamnosus* GG ATCC 53103; (**b**) *Bifidobacterium* BB-12; (**c**) *Lb. casei*; (**d**) *Lb. acidophilus*; (**e**) *Lb. plantarum* in MRS medium with different pH (2.0; 3.0; 4.0; 5.0; 6.2), (*p* < 0.05). The number of repeats for each treatment *n* = 3. Letters marked with the same color define a homogeneous group within one pH value among the tested variants of the experiment (a given color correlates with the pH value and a given letter correlates with a given homogeneous group within pH range).

Curves of changes in optical density (OD, λ = 600 nm) during culture of commercial preparations of probiotic bacteria in MRS broth with various pH values are shown on Figure 3. The viable cell counts of the tested probiotic bacteria determined by the plate method are illustrated in Table 1.

The OD of *Lb*. GG culture in MRS broth with pH 2.0 and pH 3.0 was decreasing throughout the experiment (from the initial value of ca. 0.5 to the final value of ca. 0.36) (Figure 3). The results obtained using the plate method (Table 1) indicate that during the first 12 h of the experiment at pH 2.0 or 3.0, the number of *Lb*. GG decreased to less than 4 log CFU ml^−1^. It was found that the other tested strains reacted in a similar way, except *Lb. casei* and *Lb. plantarum* in MRS with pH 3.0 (Table 1). The highest OD values were noted for this probiotic strain in MRS broth with pH 5.0 and pH 6.2 (Figure 3). The number of *Lb.* GG in the MRS with pH 5.0 after 48 h of the experiment increased by about 1.34 log order, while at the optimal pH (pH 6.2) by 2.2 log order.

The course of the growth curve plotted for the BB-12 strain at pH 4.0 indicated that the bacteria needed a lot of time to adapt to medium conditions before their cells began to divide (Figure 3). By the 36th hour of the experiment, a decrease in the number of strain BB-12 from the initial 7.13 log CFU mL^−1^ to 4.24 log CFU mL^−1^ was observed, while in the last twelve hours there was an increase in the number of bacteria by about one logarithmic order, which is also visible on the course of the OD curve (Figure 3). Only *Lb.* GG, *Lb. acidophilus* and *Lb. plantarum* strains showed an increase in cell number during incubation in MRS at pH 4.0 (Table 1).

Growth curves plotted for the BB-12 strain in MRS media with pH 5.0 and 6.2. had a similar course and indicated intensive cell proliferation (Figure 3). For all tested strains, the number of cells was increased during incubation in MRS at pH 5.0 and pH 6.2 (Table 1). The increase in the viable cell number at pH 5.0 was 1.25–3.46 log orders and at pH 6.0 about 3.0 log orders.

For most of the probiotic strains tested, the course of the growth curves was characteristic and included lag phase, log phase, and stationary phase. Worthy of notice is, however, that growth curves plotted for bacteria cultured under experimental conditions differed for each preparation (Figure 3). 

Statistical analysis showed that there was not significant difference between the OD value of *L. rhamnosus* GG growing in MRS pH 2.0, 3.0, as well as in MRS pH 5.0 and 6.2 (Figure 3). It was also shown that in the case of the BB-12 strain growing in MRS, the same homologous group was for growth in pH 2.0, 3.0 and 4.0, and, simultaneously, the OD values for this strain cultivating in MRS pH 5.0 and 6.2 belonged to the same homologous group (Figure 3). An analogous situation was observed for the MRS culture of *Lb. casei* (Figure 3). However, in the case of cultivation with the use of an *Lb. acidophilus* strain, three homologous groups were observed—the first for growth in pH 2.0 and 3.0, second for pH 4.0 and the third for growth in pH 5.0 and 6.2 (Figure 3).

Selected lag phase and log phases, initial and maximal OD values, and coefficients of the specific growth rate of the analyzed probiotic preparations are summarized in Table 2.

In the case of the *Lb. rhamnosus* GG ATCC 53103 strain, the highest value of the specific growth rate coefficient (μ = 0.084) was determined in MRS broth with pH 6.2. The μ values computed for the culture of these bacteria in MRS broth with pH 4.0 and 5.0 reached 0.042 and 0.063, respectively. In the case of MRS broth with pH 4.0, bacterial cells needed 19 h to adapt to medium conditions, whereas in MRS broth with higher pH values (5.0 or 6.2), cells of this probiotic began proliferation immediately after culture initiation. The growth curve plotted for *Lb.* GG in the medium with pH 4.0 had the longest phase of logarithmic growth of 29 h, whereas at pH 5.0 and pH 6.2, the length of this phase reached 19 and 16 h, respectively. In MRS broth with pH 4.0 and pH 5.0, the final OD value increased three times, whereas in the medium with pH 6.2 the final OD value of culture increased nearly four times in comparison with initial OD (Table 2)

The value of the μ coefficient for the growth of BB-12 strain cells increased along with increasing active acidity of the culture medium (μ = 0.093 at pH 5.0 and μ = 0.114 at pH 6.2). The growth of the cells of this strain in the medium with pH 4.0 revealed a long, nearly 40 h phase of adaptation of the cells to conditions of the medium (Figure 3). In MRS broth with pH 5.0 and 6.2, the adaptation phase lasted ca. 3 h. The length of the logarithmic growth phase noted for BB-12 in pH 5.0 was 16.5 h and in pH 6.2 was 12 h (Table 2). The highest (4.7-fold) increase in OD value of BB-12 strain culture was observed in MRS broth with pH 5.0 (Table 2).

The *Lb. casei* strain showed no growth in MRS broth with pH 4.0 (Figure 3), whereas in MRS with pH 5.0 and 6.2 the length of lag phase was the same (24.5 h). The value of μ coefficient calculated for the culture incubated at pH 6.2 was higher by 0.036 compared to the culture incubated at pH 5.0 (Table 2).

The culture of the *Lb. acidophilus* strain was characterized by the highest value of the growth rate coefficient in MRS broth with pH 6.2 (μ = 0.053). After cell introduction into the MRS broth with pH 4.0, the lag phase lasted ca. 25 h, whereas in the other media (with pH 5.0 and 6.2) it was definitely shorter and reached ca. 6 h. In MRS broth with pH 5.0, the final OD value increased 2.6-fold compared to the initial value (for comparison, in MRS broth with pH 6.2, the log phase lasted 28 h and OD increased over 4-fold) (Table 2).

No growth of the *Lb. plantarum* strain was observed in MRS broth with pH 2.0 and 3.0 (Table 1, Figure 3). The value of the μ coefficient determined for the *Lb. plantarum* strain cultured in MRS broth with pH 4.0, 5.0 and 6.2 reached 0.019, 0.039 and 0.058, respectively (Table 2). Cells of this strain started division immediately after culture onset, regardless of medium pH. The log phase lasted 29 h for the culture incubated at pH 4.0, as well as 17 and 12 h for cultures incubated at pH 5.0 and 6.2, respectively. 

### 3.2. Survivability and Growth of Bacteria Present in Commercial Probiotic Preparations in a Food Matrix Simulating Gastric Passage 

Food retention in the stomach usually lasts ca. 1–3 h [32] and liquid foods are retained. Once food has been ingested and its digestion has begun, pH value successively decreases [33,34]. 

**Figure 4:**
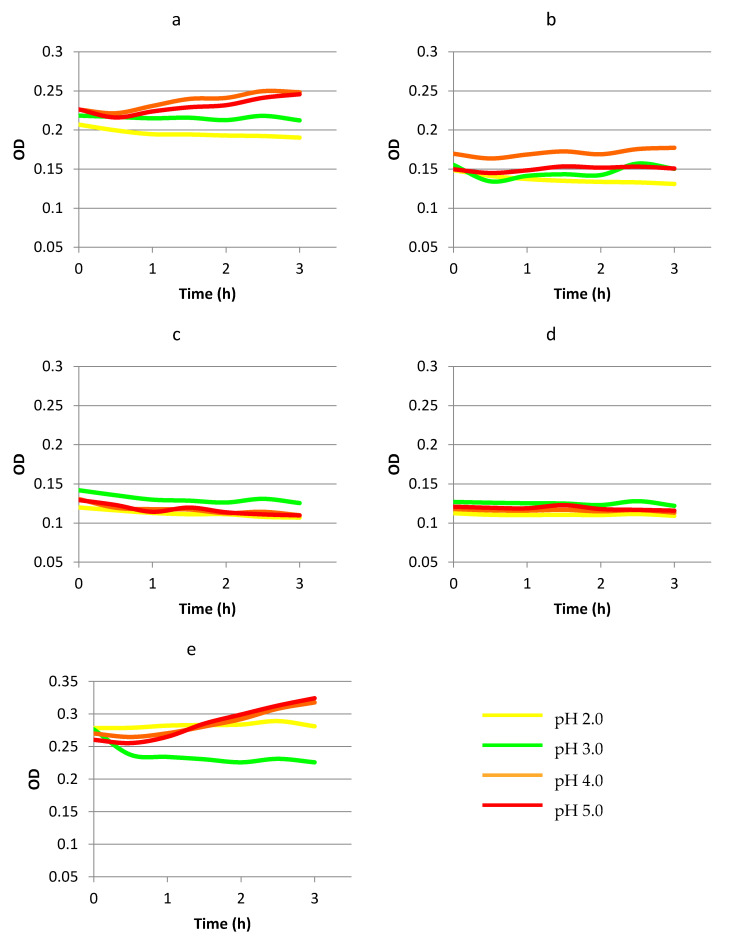
Changes in the optical density during culture of commercial probiotic strains in the food matrix under simulated condition of the gastric passage (**a**) *Lb. rhamnosus* GG ATCC 53103; (**b**) *Bifidobacterium* BB-12; (**c**) *Lb. casei*; (**d**) *Lb. acidophilus*; (**e**) *Lb. plantarum*, (*p* < 0.05). The number of repeats for each treatment *n* = 3. Letters marked with the same color define a homogeneous group within one pH value among the tested variants of the experiment (a given color correlates with the pH value and a given letter correlates with a given homogeneous group within pH range).

Curves depicting changes in OD values during the incubation of the cultures of tested preparations in the medium simulating conditions likely to occur in the stomach of a small child after consumption of a chicken–vegetable soup (CVS), and after taking a probiotic preparation in a suspension of spring water, are shown on Figure 4. Changes in the cell number of the tested strains during incubation in the gastric medium are presented in Table 3.

Gastric fluids differed in pH values, which were higher at the beginning and lower at the end of digestion. Optical density of the culture of the *Lb.* GG strain decreased insignificantly at pH 2.0 and 3.0. Already active acidity of 4.0 and 5.0 enabled the growth of these bacteria; however, in both variants of culture the OD value increased by 0.02 on average (Figure 4). A reduction in the number of *Lb.* GG cells was observed during incubation regardless of the pH of the medium. In gastric medium with pH 2.0, after just 2 h of incubation, the *Lb.* GG number decreased to less than 4 log CFU mL^−1^. BB-12, *Lb. casei* and *Lb. acidophilus* strains showed no growth during incubation, regardless of active acidity values, which was indicated by the course of curves depicting OD value changes in time of incubation (Figure 4). Among the commercial probiotic strains selected for this study, only *Lb. plantarum* showed significant growth in the GES and CSV medium at pH 4.0 and 5.0. In both cases, a 1.2-fold of increased OD (from the beginning till 3 h) was noticed. When analyzing changes in the number of cells of probiotic strains based on the results of the plate method (Table 3), it can be concluded that the *Lb. plantarum* strain was characterized by the highest resistance to low pH. In the gastric medium with pH 4.0 and pH 5.0, a slight increase in the cell number of this strain was noted (by 0.18 and 0.14 log order). In gastric medium with pH 2.0 at the last 3rd hour of the experiment, the number of *Lb. plantarum* was 4.97 log CFU mL^−1^. Similar resistance was demonstrated only for strain BB-12.

### 3.3. Survivability of Commercial Probiotic Strains in a Food Matrix Simulating Gastrointestinal Passage

**Figure 5:**
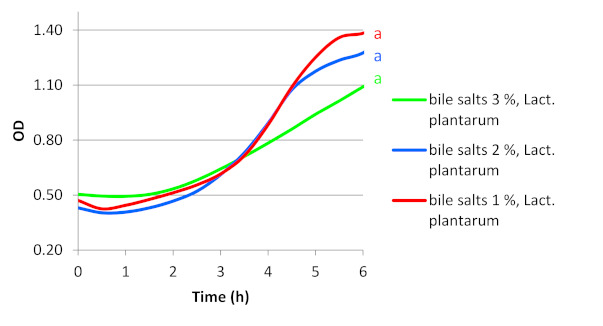
Changes in the optical density during culture of *Lb. plantarum* from commercial probiotic strains in the food matrix under simulated condition of the gastric and gastrointestinal passage; in this case it was only one homologous group signed *a* (*p* < 0.05). Each homogeneous group’s corresponding pH was marked the same color as pH line axce.

The passage of intestinal digesta through the section of the small intestine usually spans for 1–6 h [32]. No changes were observed in the optical density in any of the media simulating conditions occurring during digestion in the small intestine with BB-12, *Lb. rhamnosus*, *Lb. casei* and *Lb. acidophilus* strains (data not shown). The *Lb. plantarum* strain was the only one capable of proliferation under small intestine conditions, regardless of bile salts concentration (1%, 2% or 3%) (Figure 5). The log phases for *Lb. plantarum* took from 1.5 to 2 h for all tested concentrations of bile salts.

The analysis of the initial and final OD values of the culture of the probiotic strain *Lb. plantarum* increased about 2-fold for concentration 1% and about 3-fold for the rest of the concentrations. The adaptation phase of the *Lb. plantarum* strain reached only 1.5 h regardless of bile concentrations.

Changes in the cell number of the tested strains during incubation in the food matrix under simulated condition of the gastrointestinal passage are presented in Table 4. 

An increase in the number of cells was observed during incubation in the gastrointestinal medium irrespective of the amount of bile salt addition only in the case of *Lb. plantarum* strain. The number of *Lb. plantarum* in the gastrointestinal medium with 1% of bile after 6 h of the experiment increased by about 0.83 log order, while at 3% of bile by 0.29 order of magnitude in 1 mL. Some resistance to bile salts was found in the BB-12 strain. The final cell numbers of this strain after 6 h of incubation in gastrointestinal medium containing 1% and 2% of bile salts reached 6 log CFU mL^−1^. Only in the medium with the highest tested content of bile salts (3%) was a slight reduction in the number of cells from the initial 6.17 to 5.86 log CFU mL^−1^ determined. The most sensitive to the presence of bile salts at the level of 3% were *Lb. casei* and *Lb.* GG strains (<4 log CFU mL^−1^ from the 2nd and 4th hour of incubation, respectively).

## 4. Discussion

High variability of strains and unlimited possibilities of creating experimental conditions in scientific research significantly impair the comparison and discussion of results achieved in various studies [35]. There are several criteria, which need to be met to classify a strain as a “probiotic”. The key ones among functional criteria include tolerance to gastric juice and bile, and capability of adhesion to colonic mucosa [36,37]. Probiotic bacterial strains have to survive unfavorable conditions encountered during their gastrointestinal passage to be able to colonize the colon and to exert a positive effect on consumer/host health [32]. However, as indicated in scientific research, not all strains classified as “probiotic” meet these criteria [38,39]. Both manufacturers of probiotic preparations and pediatricians exclude the administration of a probiotic preparation in the form of a gelatin capsule to young children. Giving toddlers a capsule is not advisable because they can easily choke by swallowing it. It is strictly recommended to remove the capsule and suspend probiotic with water and administrate it in this form. For this reason described, experiments showed results of survivability of strains lacking early protection against bile salts and low pH [40,41,42,43]. 

*Lb. rhamnosus* GG is a well-characterized probiotic strain [44]. It is a commensal, which colonizes the gastrointestinal tract in humans [45]. In 1985, *Lb.* GG was patented as a probiotic partly due to its resistance to low pH and to bile salts [46]. The exact mechanism of these bacteria effecting the organism of the host remains unknown; however, bacteria of the *Lb. rhamnosus* species are implied to exhibit antimicrobial, antiviral, and diarrhea-preventing properties [47,48]. Pitino et al. [34] demonstrated that *Lb. rhamnosus* strains isolated from cheese showed high survivability in MRS broth with pH 5.0 during simulated dynamic digestion in the stomach. In our study, the *Lb.* GG strain also showed growth in MRS broth with pH 5.0 (Figure 3, Table 1). In a clinical survey conducted by Hibberd et al. [48], in 73% of volunteers to whom *Lb. rhamnosus* was administered orally in a dose of 10^10^ CFU for 28 days, reduced numbers of these bacteria were detected in feces, i.e., from 1.4 × 10^3^ to 1.3 × 10^8^ CFU [48]. Other *Lb. rhamnosus* strains isolated from wine showed growth after 24 h incubation at pH 3.5, likewise *Lb. rhamnosus* isolated from meat [49]. Goldin et al. [44] proved that *Lb. rhamnosus* GG survived incubation in the medium with pH 3.0. Results of other studies indicate high resistance of this strain to a bile salt concentration of 1.5% [49]. In our study, the *Lb. rhamnosus* GG strain showed no growth at bile salt concentrations of 1, 2 or 3% (Table 4).

Bacteria of the genus *Bifidobacterium* possess the GRAS (Generally Recognised As Safe) status and constitute part of the natural microflora of the gastrointestinal tract of humans (likewise other probiotic bacteria, e.g., those from the genus *Lactobacillus*); therefore, they are often used as components of commercial dietary supplements [3]. In our experiment, the BB-12 strain showed no growth either in MRS broth with pH 2.0 or with pH 3.0 (Figure 3b, Table 1). In MRS broth with pH 4.0, we observed the growth of BB-12 bacteria already after 36 h (Table 1). Analyses conducted in our study showed no growth of BB-12 strain under simulated gastrointestinal conditions. De Castro-Cislaghi et al. [30] observed a reduction in the cell count of the BB-12 strain in the presence of bile salts in the concentration of 1%, from the initial value of ca. 9.5 log CFU/g to ca. 9 log CFU/g after 3 h incubation. In addition, they demonstrated that resistance to various pH values and concentrations of bile salts is a variable, strain-specific feature.

Probiotic lactic bacteria of the species *Lb. casei* have been widely applied in the production of fermented foods [50]. The administration of lyophilized preparations of these bacteria is believed to reduce the blood level of cholesterol and to impair proliferation of cancer cells [51,52,53]. Apart from the *Lb. casei* strain, commercial preparation 3 tested in our study contained inulin, which was supposed to support its viability. Growth tests conducted in the model MRS culture medium demonstrated that the phase of cell adaptation to conditions of the culture medium was one of the longest in the case of this strain (ca. 25 h, Figure 3, Table 2). This was the only strain which showed no growth in MRS broth with pH 4.0 (Table 1). Cells of *Lb. casei* began to divide already at pH 5.0, although their adaptation phase was again one of the longest compared to the other strains (Figure 3, Table 2).

In our study, the *Lb. casei* strain showed no growth in conditions simulating the gastric and/or gastrointestinal passage (Table 3 and Table 4). Dimitrellou et al. [32] demonstrated successive viability loss for the *Lb. casei* strain incubated in gastric media. After 3 h of incubation, they observed a decrease in the cell count of this strain by ca. 4.0 log CFU g^−1^ at the initial pH 2.0 and by 1.5 log CFU g^−1^ at pH 3.0. In addition, they showed the presence of bile salts in the concentration of 1 gL^−1^ to evoke *Lb. casei* cell count reduction by nearly 6 log CFU g^−1^ after 6 h of incubation. In turn, Mishra and Prasad [29] proved that all seven analyzed strains of *Lb. casei* survived incubation at pH 3.0, and two of them were viable once pH was decreased to the value of 2.0. All seven analyzed strains were viable after 12 h incubation in solutions with bile salt concentrations of 1 and 2%.

Bacteria of the *Lb. acidophilus* species naturally occur in the gastric tract of humans and animals [54]. In our study, the *Lb. acidophilus* NCFM strain (preparation 4) showed no growth under experimental gastro-intestinal conditions (Table 4).

Representatives of the *Lb. plantarum* species are also implied to exhibit probiotic traits. The *Lb. plantarum* NRRL-B4496 strain is one of the main probiotics used in fermented food products [55,56]. Multiple scientific works indicate this strain to be capable of inhibiting the growth of certain pathogens that induce diseases of the alimentary tract, e.g., *Helicobacter pylori* or *Listeria monocytogenes* [56,57,58,59,60].

Commercial probiotic preparations are often supplemented with prebiotics, the task of which is to increase the chances of probiotic strains for the survival of adverse conditions during gastrointestinal passage and to sustain their metabolic activity [3,61,62]. The most frequently used prebiotics include inulin [63,64], β-glucan, and fructooligosaccharides (FOS) [62,65,66].

Obtained results clearly show that, among all analyzed commercial probiotic strains, only the *Lb. plantarum* was the most resistant to the applied conditions of the culture medium. It showed a noticeable growth under both in vitro gastric conditions at pH 4.0 and 5.0, as well as in vitro intestinal conditions at all tested concentrations of bile salts. Interestingly, its preparations did not contain a prebiotic. 

No OD changes of the tested commercial probiotics under the assumed experimental conditions does not have to indicate their incapability for surviving the in vivo gastrointestinal passage. However, obtained results from the plate count method show that the *Lb. plantarum* strain had the best capability for growth, which suggests it could proliferate in intestines also under in vivo conditions. Considering the fact that the tested probiotics were deprived of a gelatin capsule at the beginning of the experiment, it can be concluded that *Lb. plantarum* exhibited distinctive properties that allowed this strain to survive the simulated conditions of the passage. This is valuable knowledge, considering that exogenous probiotics share a limited capability for adhesion to cells of the intestinal epithelium and that their major part is excreted with feces.

The scheme of experiments, shown in this study, largely covers the simplified conditions during the gastrointestinal transit in the human body and does not include many factors. For some of the probiotic strains present in the tested preparations, there are literature data from several years ago showing their beneficial effect on the course and shortening of the duration of diarrhea of various origins [67]. The medical practice of using probiotic food supplements to children consists of administering them, for example, after or still during antibiotic therapy for the treatment of diarrheal disorders [67]. Recent data concerning the effect of probiotics on inflammation of gastroenteritis, which is often manifested by diarrhea, are different from those previously described. Freedman et al. [68] showed that administration of a probiotic product containing *Lactobacillus rhamnosus* R0011 and *L. helveticus* R0052, at a dose of 4.0 × 10^9^ CFU/unit twice daily, did not reduce the incidence of diarrhea in children with gastroenteritis. Similarly, among preschool children with acute gastroenteritis, those who had taken *L. rhamnosus* GG did not show better outcomes than those children who had received placebo [69].

## 5. Conclusions

Results of our study show that among all analyzed probiotic bacteria from commercial preparations avaliable on Polish market, the Lb. plantarum was the most resistant to the applied conditions of the experiment. It showed a noticeable growth under both in vitro gastric conditions at pH 4.0 and 5.0, as well as in vitro intestinal conditions at all tested concentrations of bile salts. Interestingly, its preparations did not contain a prebiotic. In turn, preparation 2 (Bifidobacterium BB-12) contained FOS, which could affect its capability to grow under simulated conditions of the gastric passage (pH 4.0 and 5.0) and of the gastrointestinal passage at bile salts concentration of 10 gL^−1^. The remaining preparations (preparation 3 – Lb. casei and preparation 4 – Lb. acidophilus) contained inulin which, however, didn’t influence their cell proliferation capability during incubation under experimental conditions. 

No growth of the tested commercial probiotics under the assumed experimental conditions does not have to indicate their incapability for surviving the in vivo gastrointestinal passage. However, study results show clearly that the Lb. plantarum strain had the best capability for growth, which suggests it could proliferate in intestines also under in vivo conditions. It is a valuable piece of information, considering that exogenous probiotics share a limited capability for adhesion to cells of the intestinal epithelium and that their major part is excreted with feces.

## Figures and Tables

**Table 1:** Growth of commercial probiotic strains (log CFU mL^−1^ ± SD) in MRS medium with different pH.

pH	Time of Incubation (h)				
	0	12	24	36	48
*Lb. rhamnosus* GG					
2	7.76 ± 0.31	-	-	-	-
3	7.79 ± 0.11	-	-	-	-
4	7.79 ± 0.08	8.55 ± 0.11	8.83 ± 0.05	8.68 ± 0.13	9.02 ± 0.08
5	7.8 ± 0.15	9.59 ± 0.16	9.73 ± 0.12	9.88 ± 0.05	9.14 ± 0.11
6.2	7.81 ± 0.07	10.56 ± 0.03	10.03 ± 0.14	10.74 ± 0.21	10.01 ± 0.05
*Bifidobacterium* BB-12					
2	6.74 ± 0.34	-	-	-	-
3	6.82 ± 0.11	-	-	-	-
4	7.13 ± 0.58	6.13 ± 0.17	5.93 ± 0.08	4.24 ± 0.31	5.46 ± 0.45
5	6.98 ± 0.27	8.61 ± 0.12	9.02 ± 0.20	9.57 ± 0.16	9.38 ± 0.14
6.2	7.23 ± 0.23	9.94 ± 0.11	9.98 ± 0.01	10.16 ± 0.14	10.22 ± 0.14
*Lb. casei*					
2	6.12 ± 0.16	-	-	-	-
3	6.33 ± 0.19	5.16 ± 0.07	4.29 ± 0.00	-	-
4	6.14 ± 0.05	5.67 ± 0.00	5.82 ± 0.25	5.55 ± 0.01	5.12 ± 0.09
5	6.52 ± 0.08	6.41 ± 0.08	5.62 ± 0.09	6.85 ± 0.11	8.08 ± 0.09
6.2	6.42 ± 0.11	7.02 ± 0.16	7.16 ± 0.05	8.71 ± 0.05	9.13 ± 0.08
*Lb. acidophilus*					
2	6.79 ± 0.13	-	-	-	-
3	6.89 ± 0.08	-	-	-	-
4	6.63 ± 0.11	6.51 ± 0.13	6.40 ± 0.14	6.82 ± 0.14	6.99 ± 0.21
5	6.92 ± 0.22	6.83 ± 0.02	7.94 ± 0.58	8.33 ± 0.05	8.17 ± 0.16
6.2	7.01 ± 0.22	8.64 ± 0.12	10.32 ± 0.15	10.13 ± 0.04	9.97 ± 0.14
*Lb. plantarum*					
2	6.13 ± 0.39	-	-	-	-
3	6.29 ± 0.23	5.55 ± 0.08	4.17 ± 0.08	-	-
4	6.37 ± 0.25	7.36 ± 0.08	7.98 ± 0.01	9.17 ± 0.03	9.14 ± 0.24
5	6.22 ± 0.09	8.92 ± 0.08	9.28 ± 0.12	9.91 ± 0.09	9.68 ± 0.11
6.2	6.41 ± 0.03	9.32 ± 0.13	10.16 ± 0.08	10.28 ± 0.13	9.93 ± 0.03

“-”—no growth/less than 4 log CFU mL^−^¹.

**Table 2:** Selected lag and log phase lengths, initial and final OD_600_ in log phase, and coefficient of specific growth rate for the bacteria present in commercial probiotic preparations tested in MRS medium.

Strain	Variant of Culture in MRS	Length of Lag Phase (h)	Length of Log Phase (h)	Initial OD_600_ in Log Phase	Final OD_600_ in Log Phase	Coefficient of Specific Growth Rate (μ) (h^–1^)
*Lb. rhamnosus* GG ATCC 53103	pH 4.0 pH 5.0 pH 6.2	19.0 0.5 0	29.0 19.0 16.0	0.53 0.59 0.56	1.78 1.97 2.15	0.042 0.063 0.084
*Bifidobacterium* BB-12	pH 5.0 pH 6.2	3.0 2.5	16.5 12.0	0.44 0.54	2.05 2.13	0.093 0.114
*Lb. casei*	pH 5.0 pH 6.2	24.5 24.5	35.0 20.0	0.30 0.35	1.55 1.85	0.047 0.083
*Lb. acidophilus*	pH 4.0 pH 5.0 pH 6.2	25.0 5.5 6.5	16.5 49.5 28.0	0.33 0.30 0.37	0.67 0.78 1.60	0.043 0.019 0.053
*Lb. plantarum*	pH 4.0 pH 5.0 pH 6.2	0 0 1.0	29.0 17.0 12.0	0.98 0.99 1.04	1.70 1.93 2.10	0.019 0.039 0.058

**Table 3:** Growth of commercial probiotic strains (log CFU mL^−1^ ± SD) in the food matrix under simulated condition of the gastric passage.

pH	Time of Incubation (h)			
	0	1	2	3
*Lb. rhamnosus* GG				
2.0	6.92 ± 0.17	5.19 ± 0.01	-	-
3.0	7.07 ± 0.04	5.72 ± 0.08	5.55 ± 0.17	5.63 ± 0.45
4.0	7.21 ± 0.00	6.64 ± 0.05	6.78 ± 0.13	6.33 ± 0.04
5.0	7.13 ± 0.34	6.96 ± 0.17	6.82 ± 0.09	6.80 ± 0.02
*Bifidobacterium* BB-12				
2.0	5.73 ± 0.28	4.62 ± 0.34	4.58 ± 0.05	4.70 ± 0.11
3.0	6.96 ± 0.00	7.13 ± 0.16	6.88 ± 0.00	6.86 ± 0.00
4.0	7.02 ± 0.13	6.32 ± 0.12	6.54 ± 0.15	6.38 ± 0.31
5.0	7.22 ± 0.13	7.16 ± 0.16	7.31 ± 0.03	7.18 ± 0.21
*Lb. casei*				
2.0	5.80 ± 0.08	-	-	-
3.0	6.29 ± 0.11	-	-	-
4.0	6.19 ± 0.54	5.37 ± 0.17	5.23 ± 0.14	4.22 ± 0.14
5.0	6.21 ± 0.35	5.98 ± 0.32	5.61 ± 0.17	4.92 ± 0.12
*Lb. acidophilus*				
2.0	6.24 ± 0.12	5.70 ± 0.05	5.30 ± 0.00	-
3.0	6.78 ± 0.12	5.99 ± 0.07	5.13 ± 0.05	-
4.0	6.88 ± 0.09	6.23 ± 0.13	6.19 ± 0.11	6.33 ± 0.03
5.0	7.02 ± 0.16	6.90 ± 0.12	6.96 ± 0.15	6.87 ± 0.02
*Lb. plantarum*				
2.0	6.68 ± 0.06	5.19 ± 0.08	5.22 ± 0.02	4.97 ± 0.17
3.0	7.18 ± 0.05	6.30 ± 0.16	6.41 ± 0.01	6.43 ± 0.25
4.0	7.20 ± 0.00	7.40 ± 0.12	7.32 ± 0.05	7.38 ± 0.16
5.0	7.31 ± 0.13	7.44 ± 0.01	7.27 ± 0.03	7.45 ± 0.00

“-“—no growth/less than 4 log.

**Table 4:** Survival of commercial probiotic strains (log CFU mL^−1^ ± SD) in the food matrix under simulated condition of the gastrointestinal passage.

Bile Salts (%)	Time of Incubation (h)			
	0	2	4	6
*Lb. rhamnosus* GG				
1	6.13 ± 0.12	6.08 ± 0.26	5.86 ± 0.03	5.80 ± 0.37
2	6.21 ± 0.22	5.12 ± 0.00	4.87 ± 0.18	4.42 ± 0.17
3	6.06 ± 0.01	5.02 ± 0.12	-	-
*Bifidobacterium* BB-12				
1	6.32 ± 0.15	6.40 ± 0.03	6.18 ± 0.28	6.16 ± 0.22
2	6.38 ± 0.15	6.16 ± 0.2	5.97 ± 0.15	6.08 ± 0.12
3	6.17 ± 0.05	5.93 ± 0.09	5.90 ± 0.23	5.86 ± 0.06
*Lb. casei*				
1	5.30 ± 0.03	5.21 ± 0.14	4.44 ± 0.01	-
2	5.26 ± 0.12	5.07 ± 0.23	4.04 ± 0.00	-
3	5.01 ± 0.09	-	-	-
*Lb. acidophilus*				
1	6.65 ± 0.05	5.37 ± 0.00	5.16 ± 0.17	-
2	6.48 ± 0.03	5.02 ± 0.12	4.86 ± 0.03	4.71 ± 0.18
3	6.52 ± 0.22	5.12 ± 0.05	4.70 ± 0.05	-
*Lb. plantarum*				
1	5.63 ± 0.12	5.79 ± 0.28	5.84 ± 0.00	6.47 ± 0.02
2	5.72 ± 0.12	5.20 ± 0.02	5.63 ± 0.01	5.99 ± 0.33
3	5.48 ± 0.10	5.53 ± 0.01	5.70 ± 0.13	5.77 ± 0.00

“-”—no growth/less than 4 log.

## Data Availability

The data presented in this study are available on request from the corresponding author.
